# Screening of Human Tumor Antigens for CD4^+^ T Cell Epitopes by Combination of HLA-Transgenic Mice, Recombinant Adenovirus and Antigen Peptide Libraries

**DOI:** 10.1371/journal.pone.0014137

**Published:** 2010-11-30

**Authors:** Wolfram Osen, Sabine Soltek, Mingxia Song, Barbara Leuchs, Julia Steitz, Thomas Tüting, Stefan B. Eichmüller, Xuan-Duc Nguyen, Dirk Schadendorf, Annette Paschen

**Affiliations:** 1 Clinical Cooperation for Unit Dermato-Oncology, German Cancer Research Center, Heidelberg, Germany; 2 Infection and Cancer Program, German Cancer Research Center, Heidelberg, Germany; 3 Department of Dermatology, University Hospital Essen, Essen, Germany; 4 Institute of Laboratory Animal Science and Experimental Surgery, RWTH-Aachen University, Aachen, Germany; 5 Department of Dermatology, University of Bonn, Bonn, Germany; 6 Institute for Immunology and Transfusion Medicine, Mannheim, Germany; Universidade de São Paulo, Brazil

## Abstract

**Background:**

As tumor antigen-specific CD4^+^ T cells can mediate strong therapeutic anti-tumor responses in melanoma patients we set out to establish a comprehensive screening strategy for the identification of tumor-specific CD4^+^ T cell epitopes suitable for detection, isolation and expansion of tumor-reactive T cells from patients.

**Methods and Findings:**

To scan the human melanoma differentiation antigens TRP-1 and TRP-2 for HLA-DRB1*0301-restricted CD4^+^ T cell epitopes we applied the following methodology: Splenocytes of HLA-DRB1*0301-transgenic mice immunized with recombinant adenovirus encoding TRP-1 (Ad5.TRP-1) or TRP-2 (Ad5.TRP-2) were tested for their T cell reactivity against combinatorial TRP-1- and TRP-2-specific peptide libraries. CD4^+^ T cell epitopes thus identified were validated in the human system by stimulation of peripheral blood mononuclear cells (PBMC) from healthy donors and melanoma patients. Using this strategy we observed that recombinant Ad5 induced strong CD4^+^ T cell responses against the heterologous tumor antigens. In Ad5.TRP-2-immunized mice CD4^+^ T cell reactivity was detected against the known HLA-DRB1*0301-restricted TRP-2_60–74_ epitope and against the new epitope TRP-2_149–163_. Importantly, human T cells specifically recognizing target cells loaded with the TRP-2_149–163_-containing library peptide or infected with Ad5.TRP-2 were obtained from healthy individuals, and short term *in vitro* stimulation of PBMC revealed the presence of epitope-reactive CD4^+^ T cells in melanoma patients. Similarly, immunization of mice with Ad5.TRP-1 induced CD4^+^ T cell responses against TRP-1-derived peptides that turned out to be recognized also by human T cells, resulting in the identification of TRP-1_284–298_ as a new HLA-DRB1*0301-restricted CD4^+^ T cell epitope.

**Conclusions:**

Our screening approach identified new HLA-DRB1*0301-restricted CD4^+^ T cell epitopes derived from melanoma antigens. This strategy is generally applicable to target antigens of other tumor entities and to different HLA class II molecules even without prior characterization of their peptide binding motives.

## Introduction

Tumors, as altered self, express a protein repertoire different from normal cells that can be specifically recognized by T lymphocytes of the host's immune system. Accordingly, infiltration of tumors by T lymphocytes has been demonstrated for different tumor entities to be associated with improved prognosis [1]–[4], suggesting that tumor antigen-specific T cell responses have a strong impact on the outcome of the disease. Of the two tumor-specific T lymphocyte subsets, CD8^+^ T cells recognize tumor antigen-derived peptides in the context of MHC class I molecules whereas CD4^+^ T cells respond to peptide-MHC class II complexes. Due to their capability to directly kill malignant cells, cytotoxic CD8^+^ T lymphocytes (CTL) have long been defined as the ultimate effector cells in anti-tumor immunity. Indeed, adoptive transfer of tumor antigen-specific autologous CTL improved the clinical outcome of stage IV melanoma patients [5]–[7]. However, the beneficial potential of adoptively transferred T cells turned out to be more pronounced if autologous tumor infiltrating lymphocytes (TIL) instead of isolated CTL clones were administered to patients, an effect that was attributed to tumor-specific CD4^+^ T cells present among the TIL infused [8]–[10]. In fact, evidence is accumulating that CD4^+^ T cells can actually induce strong anti-tumor immune responses, as recently demonstrated in mice and humans [11]–[16].

How CD4^+^ T cells mediate anti-tumor immunity is still under investigation, but it appears that the underlying mechanisms are multiple. It is well accepted that tumor-specific CD4^+^ T cells essentially sustain the anti-tumor activity of CTL by licensing dendritic cells (DC) to effectively prime CTL [17], [18] or by maintaining profound CTL memory [19], as well as by direct stimulation of CTL [20]. Furthermore, recent studies demonstrate that adoptively transferred CD4^+^ T cells can induce tumor rejection also independently of CD8^+^ T cells. This indirect process was shown to be based on the release of cytokines by CD4^+^ T cells [12] and on the CD4^+^ T cells' interaction with other immune cells such as macrophages and NK cells [21]. Notably, Quezada et al. and Xie et al. recently showed that naïve tumor antigen-specific CD4^+^ T cells, upon adoptive transfer into lymphopenic mice, can even differentiate into cytotoxic T cells that eradicate large established tumors [11], [14]. These results are also in accordance with a clinical study describing the complete remission of stage IV metastatic melanoma upon adoptive transfer of *ex vivo* expanded autologous tumor antigen-specific CD4^+^ T cells [15].

Thus, tumor antigen-specific CD4^+^ T cells essentially contribute to anti-tumor immunity which has strongly stimulated the interest in the identification of their target epitopes. The widely applied “reverse immunology” approach for epitope identification is based on *in silico* prediction of antigen-derived peptides with high binding affinities to a specific MHC molecule. The candidate sequences are then synthesized and loaded onto DC for *in vitro* priming of autologous CD4^+^ T cells. Finally, peptide-reactive T cells are employed to demonstrate generation and presentation of the corresponding epitope by antigen-loaded target cells. Unfortunately, most allele-specific peptide binding motifs of MHC class II molecules are highly degenerated making the algorithm-based *in silico* prediction of potential CD4^+^ T cell epitope sequences still speculative. Thus, after extensive T cell culture only a subgroup of predicted peptides can be verified as natural epitopes. Though different CD4^+^ T cell epitopes from tumor antigens such as CEA [22], p53 [23] or TRP-2 [24] have been defined by *in silico* prediction, we set out to overcome the drawbacks of this strategy by setting up a comprehensive screening approach based on the immunization of HLA-transgenic mice with recombinant adenovirus encoding human melanoma antigens and subsequent scanning of the T cell responses *in vitro* with the help of combinatorial antigen-specific peptide libraries. This approach allowed us to directly concentrate our efforts on naturally processed tumor antigen-specific epitopes presented by a given MHC class II restriction element. As target antigens the melanoma differentiation antigens TRP-1 and TRP-2 were chosen, since both proteins are known to be frequently recognized by CD8^+^ T cell in melanoma patients [25], [26]. In fact, targeting of TRP-1 by adoptively transferred CD4^+^ T cells was recently shown to mediate eradication of large established tumors in mice [14].

## Materials and Methods

### Ethics Statement

All studies on human material were approved by the institutional review board of the University Medicine Mannheim (Mannheim, Germany). Blood samples from healthy donors and melanoma patients were collected upon written consent. Animal experiments were approved by the internal ethics committee of the German Cancer Research Center and by the District Government in Karlsruhe, Germany (approval ID 35-9185.81/G-86/04).

### HLA-DRB1*0301-transgenic mice

HLA-DRB1*0301-transgenic (HLA-DR3tg) mice expressing the HLA-DRB1*0301 molecule on an IA^0/0^ H2 background were kindly provided by Chella David (Mayo Medical School, Rochester MN) [27]. Transgene expression was confirmed by flow cytometric analysis of peripheral blood lymphocytes (PBL) stained with a FITC-conjugated, pan HLA-DR-specific monoclonal antibody L243 (Becton Dickinson, Heidelberg, Germany). Mice were housed under SPF conditions in individually ventilated cages within the animal facility of the German Cancer Research Center.

### Peptide libraries and synthetic candidate peptides

**Figure 1:**
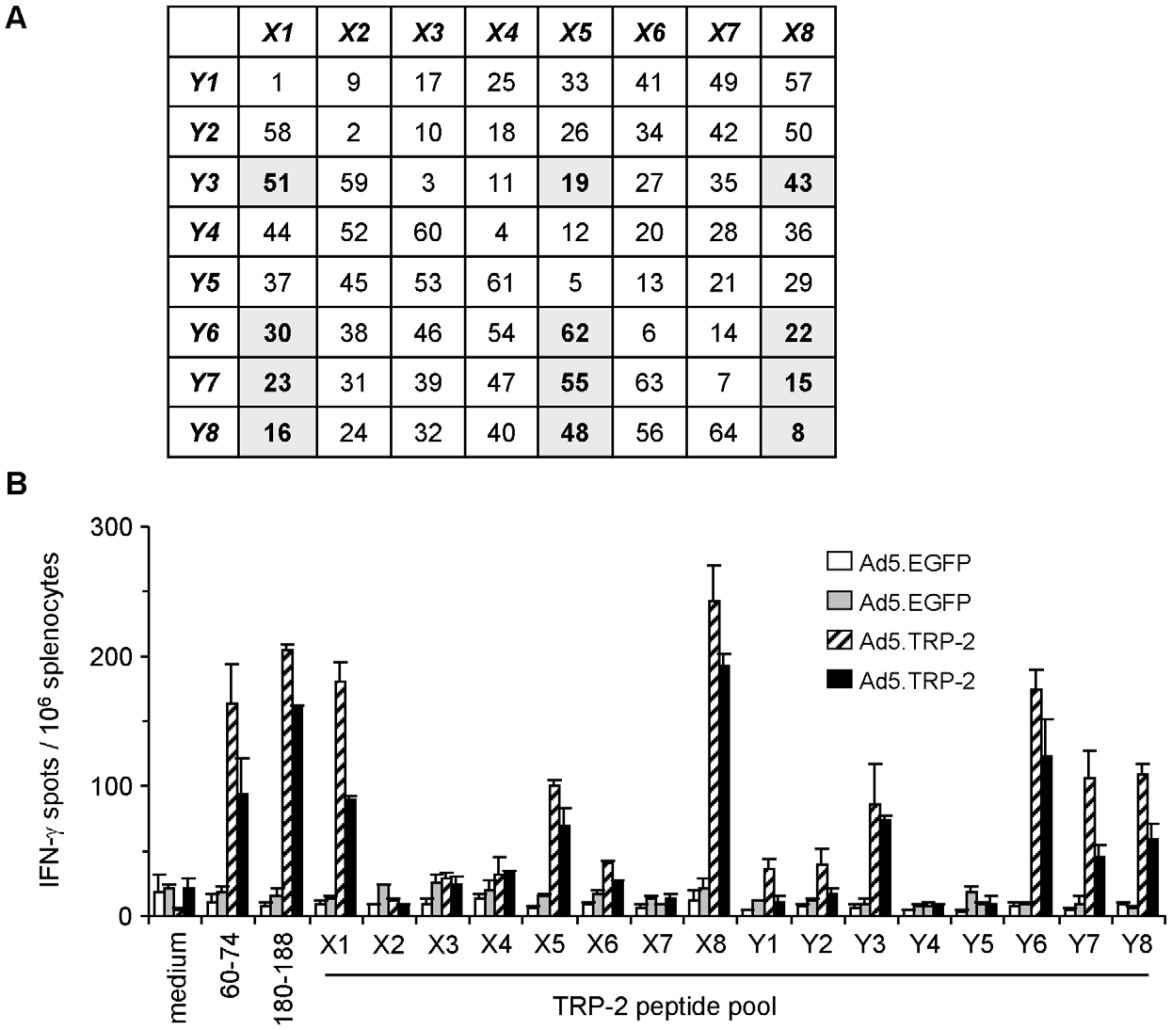
Combinatorial peptide library screening allows detection of individual library peptides containing TRP-2-specific T cell epitopes. *A*, Composition of the TRP-2-specific library peptide pools X1 to X8 and Y1 to Y8 used for combinatorial screening of specific T cell responses *ex vivo*. Individual library peptides determined by combinatorial screening are highlighted. *B*, Spleen cells from HLA-DR3tg mice injected i.p. with 5×10^8^ pfu Ad5.TRP-2 or Ad5.EGFP (2 mice per group) were screened *ex vivo* by IFN-γ ELISpot assay for recognition of TRP-2-specific library peptide pools. T cell responses of two control mice (Ad5.EGFP) and two TRP-2-immunized mice (Ad5.TRP-2) are represented as individual columns in the diagram. Reactivity against two controls, the H2-K^b^-restricted CD8^+^ T cell epitope TRP-2_180-188_ and the HLA-DRB1*0301-restricted CD4^+^ T cell epitope TRP-2_60–74_ was tested in addition. Error bars show standard error of the mean. Experiments were performed four times, yielding similar results.

TRP-1- and TRP-2-specific peptide libraries consisting of 66 and 64 peptides (20mers) respectively, overlapping by 12 amino acids, were purchased from JPT (JPT Peptide Technologies, Berlin, Germany) and Mimotopes (Mimotopes, Clayton, Australia). Library peptides were resolved in DMSO at a concentration of 20 or 50 mg/ml and were kept frozen at −20°C until use. Combinatorial peptide libraries specific for TRP-1 and TRP-2 were established by setting up 16 library peptide pools (X1–X8 and Y1–Y8), each pool consisting of eight peptides combined in such a way that each individual library peptide was shared once by two particular pools (Fig. 1A) [28]. The two C-terminal peptides of the TRP-1-specific peptide library corresponding to TRP-1_513–532_ and TRP-1_518–537_ (peptides #65 and #66) respectively, were included as separate peptides in the assays. Candidate peptides selected by library screening were synthesized by Fmoc chemistry and were FPLC-purified at the peptide synthesis core facility of the DKFZ.

### Recombinant adenoviruses

E1- and E3-deleted replication-deficient, recombinant adenoviruses encoding human TRP-2 (Ad5.TRP-2) or EGFP (Ad5.EGFP) were described previously [29], [30]. The adenoviral vector expressing human TRP-1 (Ad5.TRP-1) was generated through Cre-lox recombination as described for Ad5.TRP-2 [29]. TRP-1 expression was verified by indirect immunostaining of Ad5.TRP-1-infected human HEK293T cells (multiplicity of infection (MOI)  = 1) using supernatant of the TRP-1-specific hybridoma TA99, kindly provided by Alan N. Houghton (Sloan Kettering-Institute, New York, NY), together with a goat anti-mouse-Cy3 conjugate (BD Pharmingen, Heidelberg, Germany). Recombinant adenoviruses were amplified in HEK293T cells and were subsequently purified by CsCl gradient centrifugation as described before [29].

### Immunization of HLA-DR3tg mice and analysis of murine T cell responses *in vitro*

Mice were immunized by intraperitoneal (i.p.) injection of 5×10^8^ plaque forming units (pfu) Ad5.TRP-2 or Ad5.TRP-1. As a control, Ad5.EGFP was injected at the same dose. Two weeks later, spleen cells from immunized mice were tested for *ex vivo* recognition of TRP-2- or TRP-1-specific peptide library pools by interferon (IFN)-γ ELISpot assays using αMEM (Sigma) supplemented with 10% FCS (Biochrom, Berlin, Germany), 2 mmol/l glutamine (Gibco-Invitrogen, Karlsruhe, Germany), and 50 µmol/l 2-mercaptoethanol. The pool concentration of each library peptide was 1.5 µg/ml. The same concentrations were used when TRP-2-specific library peptides were screened individually, whereas individual TRP-1 library peptides were used at a concentration of 5 µg/ml. For *in vitro* expansion of peptide-specific T cells, splenocytes from immunized mice were incubated in the presence of the selected peptides (5 µg/ml). After 6 days, 2.5% (v/v) supernatant of conA-stimulated rat spleen cell cultures was added as a source of interleukin (IL)-2. Two days later, T cell responses against the cognate peptides were analyzed by intracellular IFN-γ staining, using a cytokine staining kit (BD Pharmingen) according to the manufacturer's instructions in combination with goat anti-mouse CD4- and CD8-specific monoclonal antibody conjugates (BD Pharmingen).

### IFN-γ ELISpot assays with murine spleen cells

IFN-γ ELISpot assays were performed using Multiscreen ELISpot plates (Millipore, Schwalbach, Germany) coated with 1 µg/ml rat anti-mouse IFN-γ capture antibody (R4-6A2; Becton Dickinson) for 1 to 2 h at 37°C. After blocking, 10^6^ spleen cells were co-incubated together with library peptide pools or individual peptides in a total volume of 200 µl per well for 16–18 h in the above mentioned medium. After washing, wells were incubated with 2 µg/ml biotinylated rat anti-mouse IFN-γ antibody (XMG1.2; Becton Dickinson) for 2 h at 4°C. Again after washing, plates were incubated with avidin-conjugated alkaline phosphatase (Becton Dickinson) for 30 min. IFN-γ-specific spots were developed by adding 100 µl BCTP/NBT (Sigma, Deisenhofen, Germany) into each well. Reaction was stopped after 2–4 min by rinsing the wells with distilled water. Spots were counted using an ELISpot reader (AID, Strassberg, Germany).

### Detection of peptide-specific human T cell responses

The HLA genotype of PBMC from healthy donors and patients was determined by high-resolution PCR typing. For analysis of specific T cell responses, frozen PBMC from HLA-DRB1*0301^+^ healthy donors and HLA-DRB1*03^+^ patients were thawed and cultured overnight in T cell medium consisting of RPMI 1640/HEPES/2 mM glutamine supplemented with 10% human AB serum (PAA Laboratories), 100 U/ml penicillin, 100 µg/ml streptomycin. Total PBMC or PBMC depleted of CD25^+^ T cells with anti-CD25-coated microbeads (Miltenyi Biotec, Bergisch Gladbach, Germany) according to the manufacturer's instructions, were seeded at 2×10^6^ cells/ml per well of a 24-well plate in T cell medium containing 20 IU/mL IL-2 and 10 ng/mL IL-7. Peptides were added at a concentration of 10 µg/ml, control cells were left without peptide. Half of the supernatant was exchanged every 4 days. After 17 and 25 days, cells were harvested and screened for peptide-specific reactivity in an IFN-γ ELISpot assay as described before [31]. Briefly, 10^5^ PBMC/well were incubated with peptide (5 µg/ml) in a 96-well microfiltration plate coated with anti-IFN-γ mAb (1-D1K; Mabtech, Stockholm, Sweden). After 16 h, secondary biotinylated anti-human IFN-γ mAb (7-B6-1; Mabtech) was added to the wells and captured cytokine was detected with avidin-peroxidase (Vectastain Elite Kit; Vector Laboratories, Burlingame, CA) and AEC substrate (Sigma, Deisenhofen, Germany). Spots were imaged and counted using the ELISpot Bioreader 3000 (Bio-Sys, Karpen, Germany). All determinations were performed at least in duplicates. The data are presented as mean numbers of IFN-γ spots per 10^5^ cells.

### Generation of peptide-specific human T cell lines and specificity analysis

T cells specific for TRP-2 peptide #19 were primed *in vitro* by stimulation of total PBMC from healthy individuals with autologous peptide-loaded DC and repeated restimulation as described previously [24]. Peptide-stimulated CD4^+^ T cells were purified with anti-CD4-specific mAb coated microbeads (Miltenyi Biotec), according to the manufacturer's protocol. Reactivity of CD4^+^ T cells (10^5^) was determined against peptide-loaded or adenovirus-infected target (stimulator) cells (5×10^4^) in IFN-γ ELISpot assays as described above. For peptide loading, stimulators (T2.DR3 cells [24], kindly provided by Frank Momburg, German Cancer Research Center, Heidelberg, Germany) were incubated with 5 µg/ml peptide for 2 h. To obtain virus-infected target cells, autologous PBMC depleted of CD3^+^ T cells (CD3^−^ PBMC) with anti-CD3-specific mAb coated microbeads (Miltenyi Biotec) were infected overnight with Ad5.TRP-2 or Ad5.EGFP (MOI = 100). All determinations were done at least in duplicates. The data are presented as mean spot numbers per 10^5^ responder cells.

CD4^+^ T cells specific for TRP-1_284–298_ were obtained after short term peptide stimulation of PBMC from patient VHP. Therefore, PBMC were stimulated once with peptide TRP-1_284–298_ (10 µg/ml) and seeded at 2×10^6^ cells/ml per well of a 24-well plate in T cell medium plus cytokines. After 25 days, CD4^+^ T cells were purified from PBMC and reactivity of T cells against peptide-loaded T2.DR3 target cells or infected autologous CD3^−^ PBMC was determined, as described above. Furthermore, responses of TRP-1_284–298_-specific CD4^+^ T cells to the following melanoma cell lines were tested: Ma-Mel-103b (HLA-DRB1*0301^+^, TRP-1^−^), Ma-Mel-108 (HLA-DRB1*0301^+^, TRP-1^+^), Ma-Mel-153 (HLA-DRB1*0301^−^, TRP-1^+^) as well as against virus-infected Ma-Mel-103b cells. TRP-1 and HLA-DR expression by melanoma cells was determined by quantitative RT-PCR and flow cytometry, respectively (data not shown).

## Results

### Screening for TRP-2-specific T cell responses in Ad5.TRP-2-immunized HLA-DR3tg mice

Applying the “reverse immunology” approach we previously identified TRP-2_60–74_ as a HLA-DRB1*0301-restricted CD4^+^ T cell epitope [24]. In order to establish a more comprehensive screening strategy, we developed an approach based on the antigen-specific immunization of HLA-DR3tg mice with recombinant adenovirus for *in vivo* induction of strong CD4^+^ T cell responses that were subsequently analyzed *in vitro* for their specificity against a peptide library covering the entire antigen sequence.

Therefore, HLA-DR3tg mice were injected with recombinant adenovirus encoding human TRP-2 (Ad5.TRP-2) or with recombinant adenovirus encoding EGFP (Ad5.EGFP). After 14 days, spleen cells from immunized mice were analyzed *ex vivo* in an IFN-γ ELISpot assay for their reactivity against a TRP-2-specific peptide library. The library consisted of a total of 64 overlapping 20mer peptides, covering the complete TRP-2 protein sequence. These peptides were combined in 16 peptide pools X1–X8 and Y1–Y8, whose composition was designed in a way that each pool consisted of 8 peptides and that each individual peptide was shared by two particular pools, allowing rapid identification of the epitope containing library peptide (Fig. 1A) [28]. We found that the TRP-2 peptide pools X1, X5 and X8 together with Y3, Y6, Y7 and Y8 were recognized by splenocytes from Ad5.TRP-2-immunized mice, whereas no reactivity was observed in case of Ad5.EGFP-treated animals (Fig. 1B). The twelve library peptides shared by the recognized pools, highlighted in Fig. 1A, were thus considered as potential epitope containing candidates and were subjected to further analysis. Notably, mice injected with Ad5.TRP-2 recognized two control peptides, the H2-K^b^-restricted CD8^+^ T cell epitope TRP-2_180–188_ [32] and the HLA-DRB1*0301-restricted CD4^+^ T epitope TRP-2_60–74_ [24], demonstrating that immunization of mice with Ad5.TRP-2 should provide a solid basis for the identification of yet unknown T cell epitopes.

### Identification of TRP-2-derived CD4^+^ T cell epitopes in HLA-DR3tg mice

**Figure 2:**
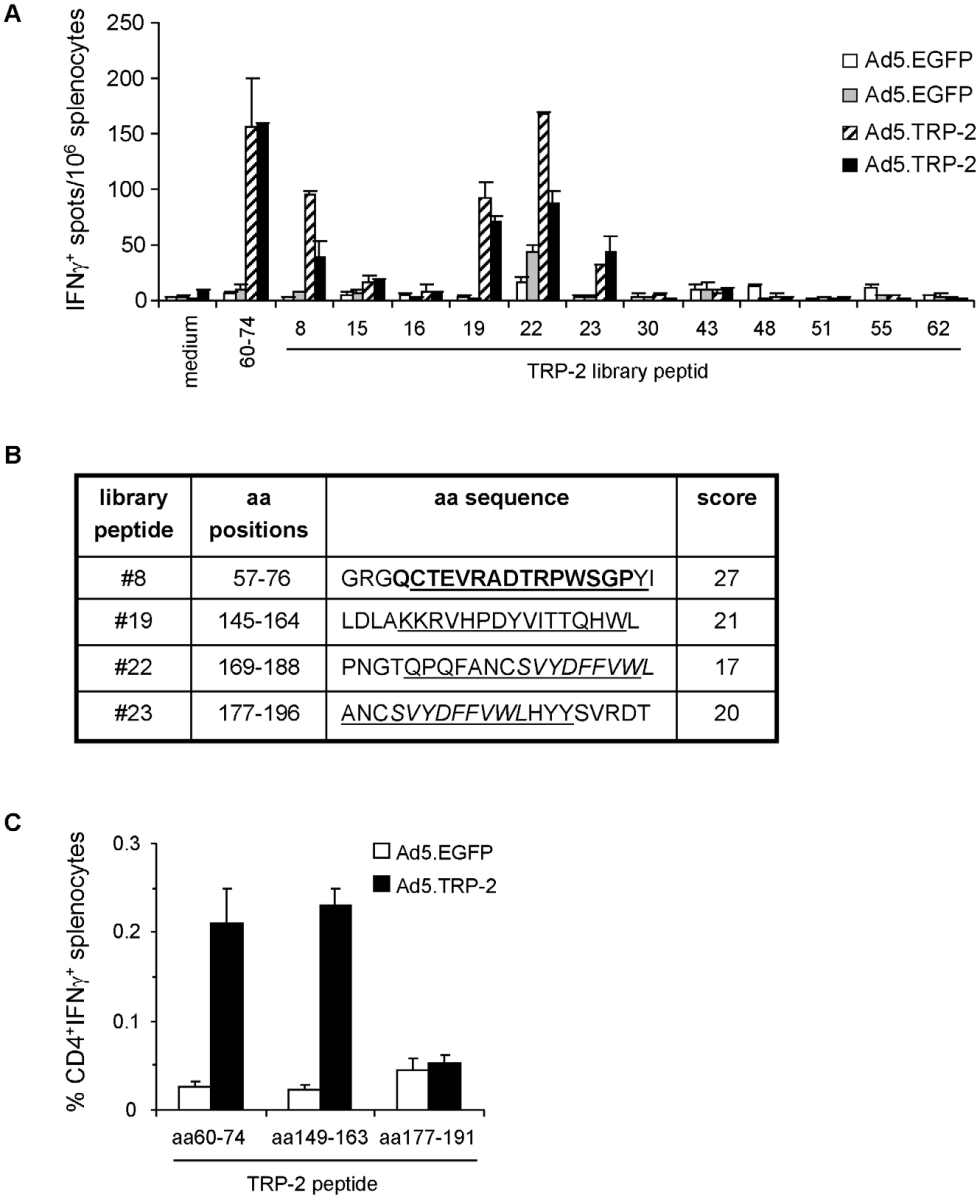
Phenotypic analysis of TRP-2-specific T cell responses induced in Ad5.TRP-2-immunized HLA-DR3tg mice. *A*, Spleen cells from HLA-DR3tg mice injected i.p. with 5×10^8^ pfu Ad5.TRP-2 or Ad5.EGFP (2 mice per group) were screened *ex vivo* by IFN-γ ELISpot assay for reactivity against individual TRP-2-specific library peptides determined by combinatorial analysis. T cell responses of two control mice (Ad5.EGFP) and two TRP-2-immunized mice (Ad5.TRP-2) are represented as individual columns in the diagram. Error bars show standard error of the mean. Experiments were performed three times, yielding similar results. *B*, Selected TRP-2-derived library peptides #8, #19, #22 and #23 are indicated by amino acid (aa) positions and aa sequence. Peptides were analyzed *in silico* by the SYFPEITHI algorithm [33] for the presence of predicted HLA-DRB*0301 binding sequences (underlined). Prediction scores for HLA-DRB1*0301-restricted epitopes are listed on the right. Known HLA-DRB1*0301-restricted CD4^+^ T cell epitopes are typed in bold and the H2-K^b^-restricted CTL epitope TRP-2_180–188_ is written in italics. *C*, HLA-DR3tg mice received i.p. injections of 5×10^8^ pfu Ad5.TRP-2 or Ad5.EGFP (3 mice per group). Two weeks later spleen cells from infected mice were harvested and stimulated *in vitro* with the indicated peptides. After 6 days, splenocyte cultures were analyzed for the presence of peptide-reactive T cells by intracellular IFN-γ staining. Stained cells were analyzed by flow cytometry for the percentage of IFN-γ^+^ CD4^+^ T cells. Error bars show standard error of the mean of three immunized mice. Experiments were performed three times yielding similar results.

In a subsequent experiment the twelve candidate peptides determined above were tested individually for their recognition by splenocytes from Ad5.TRP-2-immunized HLA-DR3tg mice (Fig. 2A). We found that the four candidate peptides (#8, #19, #22, #23) could stimulate IFN-γ secretion by splenocytes from Ad5.TRP-2-immunized recipients but not from Ad5.EGFP-treated animals. Thus, the number of potential epitope containing library peptides could be minimized from initially 64 to 4 by two sequential immunization steps.

The four library peptides #8, #19, #22 and #23 were subsequently entered into the SYFPEITHI data base for prediction of potential HLA-DRB1*0301- and H2-K^b^-restricted CD4^+^ and CD8^+^ T cell epitopes, respectively [33]. Library peptide #8 was proposed to represent a CD4^+^ T cell target sequence corresponding to the known TRP-2_60–74_ epitope, previously identified in our own studies (Fig. 2B). Peptide #19 was predicted to harbour a so far unknown HLA-DRB1*0301-restricted epitope TRP-2_149–163_. Library peptides #22 and #23 appeared as particular candidates since these peptides containing the known H2-K^b^-restricted CTL epitope TRP-2_180–188_ were also predicted to cover a CD4^+^ T cell epitope (TRP-2_177–191_). Notably, the data base predicted thirteen additional candidates as potential HLA-DRB1*0301-restricted epitopes with relatively high prediction scores of >21 (not shown). However, except for peptide TRP-2_60–74_ none of the library peptides containing the respective predicted epitope sequences was recognized by the spleen cells of immunized HLA-DR3tg mice (Fig. 1 and data not shown).

In order to prove recognition of the candidate peptides by CD4^+^ T cells, shortened synthetic 15mers derived from the 20mer library peptides were used to restimulate splenocytes from Ad5.TRP-2-immunized HLA-DR3tg mice *in vitro*. Performing intracellular IFN-γ stainings it was found that peptides TRP-2_60–74_ and TRP-2_149–163_ derived from library peptides #8 and #19, respectively, clearly induced IFN-γ secretion by CD4^+^ spleen cells (Fig. 2C). Among total spleen cells of Ad5.TRP-2-immunized mice 0.21% and 0.23% CD4^+^ IFN-γ^+^ T cells were detected on average upon stimulation with TRP-2_60–74_ and TRP-2_149–163_, respectively, whereas only around 0.025% double positive spleen cells were found in cultures from control mice. In contrast, library peptides #22 and #23 induced a profound CD8^+^ T cell response, most likely due to the CTL epitope TRP-2_180–188_ contained within these peptides, but failed to stimulate CD4^+^ T cells (Fig. 2B, data not shown). Thus, applying the combinatorial antigen-specific peptide library screening approach, peptide #19 was found to contain the new TRP-2_149–163_ epitope recognized by CD4^+^ T cells from HLA-DR3tg mice.

### CD4^+^ T cells from melanoma patients respond to the TRP-2_149–163_ epitope

**Figure 3:**
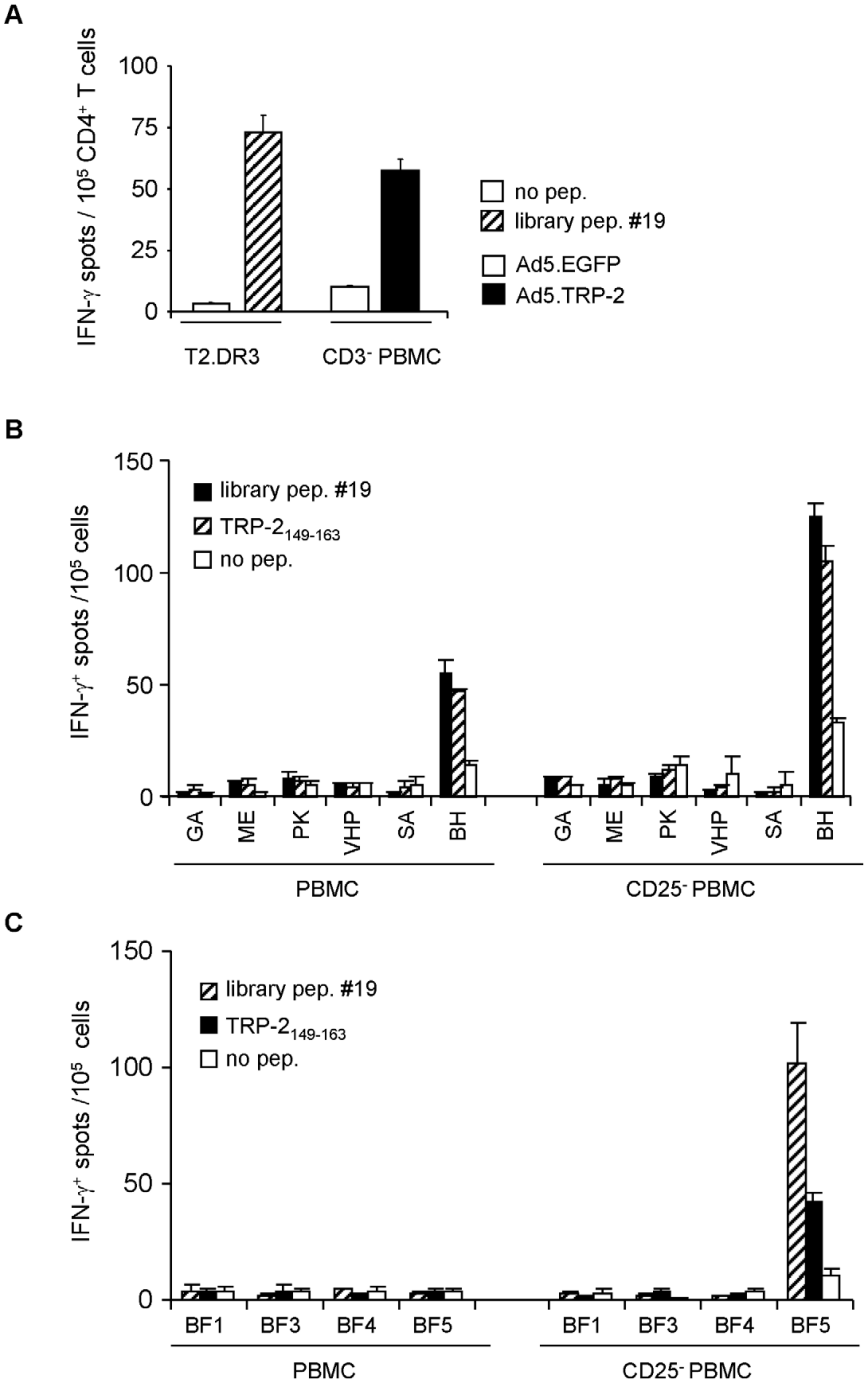
CD4^+^ T cells from melanoma patients respond to the HLA-DRB1*0301-restricted epitope TRP-2_149–163_. *A*, PBMC from a HLA-DRB1*0301^+^ healthy donor were primed *in vitro* with autologous DC pulsed with library peptide #19. After two rounds of *in vitro* restimulation, CD4^+^ T cells were tested against T2.DR3 target cells pulsed with library peptide #19 and for recognition of autologous CD3^+^-depleted PBMC (CD3^−^ PBMC) infected with Ad5.TRP-2 or with control virus. One representative experiment out of two is presented. *B*, Total PBMC (left panel) or CD25^+^-depleted PBMC (right panel) from six HLA-DRB1*03^+^ melanoma patients and *C*, four HLA-DRB1*0301^+^ healthy donors were incubated *in vitro* with library peptide #19. After 17 days, cells were tested by IFN-γ ELISpot assay for their reactivity against library peptide #19 or the epitope TRP-2_149–163_. All determinations were performed at least in duplicates. Data are presented as mean numbers of IFN-γ spots per 10^5^ cells. Error bars show standard error of the mean.

Next, we asked whether human CD4^+^ T cells would also respond to library peptide #19. Therefore, we set out to generate a peptide #19-specific CD4^+^ T cell line by *in vitro* priming of T cells from a HLA-DRB1*0301^+^ healthy donor with peptide-loaded autologous DC. After the second *in vitro* restimulation, CD4^+^ T cell specificity was tested against T2.DR3 cells loaded with peptide #19 versus non-loaded target cells. Performing IFN-γ ELISpot assays we observed that the CD4^+^ T cells responded to T2.DR3 target cells only in the presence of peptide #19 (Fig. 3A). Importantly, these T cells reacted specifically also against autologous stimulator cells infected with Ad5.TRP-2 but not against Ad5.EGFP-infected stimulators (Fig. 3A). Thus, we concluded that peptide #19, identified as a CD4^+^ T cell epitope containing library peptide in HLA-DR3tg mice, also provides a naturally processed epitope in humans.

Based on this result we screened PBMC from six HLA-DRB1*03^+^ melanoma patients (GA, ME, PK, VHP, SA, BH) and four HLA-DRB1*0301^+^ healthy donors (BF1, BF3, BF4, BF5) for the presence of peptide #19-reactive T cells. Therefore, PBMC stimulated once *in vitro* with peptide #19 for 17 days were tested for their reactivity against library peptide #19 in comparison to the shortened variant TRP-2_149–163_. Interestingly, PBMC from patient BH showed specific recognition of both peptides, whereas none of the healthy donors reacted specifically under these conditions (Fig. 3B, C; left panel).

Since depletion of CD25^+^ regulatory T cells (Treg) from PBMC has been demonstrated to enhance detection of antigen-specific CD4^+^ T cell responses [34], we additionally stimulated CD25-depleted PBMC from melanoma patients and healthy individuals with library peptide #19. Remarkably, detection of peptide #19 reactivity was very much improved, reflected by the enhanced IFN-γ response of CD4^+^ T cells from patient BH and by the fact that peptide specific T cells now became detectable also in case of healthy donor BF5 (Fig. 3B, C; right panel). In all cases, T cell cultures recognizing library peptide #19 showed cross-reactivity also against the shortened peptide variant TRP-2_149–163_. Similarly, cross-reactivity was observed when peptide TRP-2_149-163_ was used for primary *in vitro* stimulation of PBMC (data not shown). Thus, peptides #19 and TRP-2_149–163_ can stimulate specific CD4^+^ T cells that are mutually cross-reactive, suggesting that TRP-2_149–163_ is a HLA-DRB1*0301-restricted T cell epitope also in humans.

### Screening for TRP-1-specific T cell responses in Ad5.TRP-1-immunized HLA-DR3tg mice

**Figure 4:**
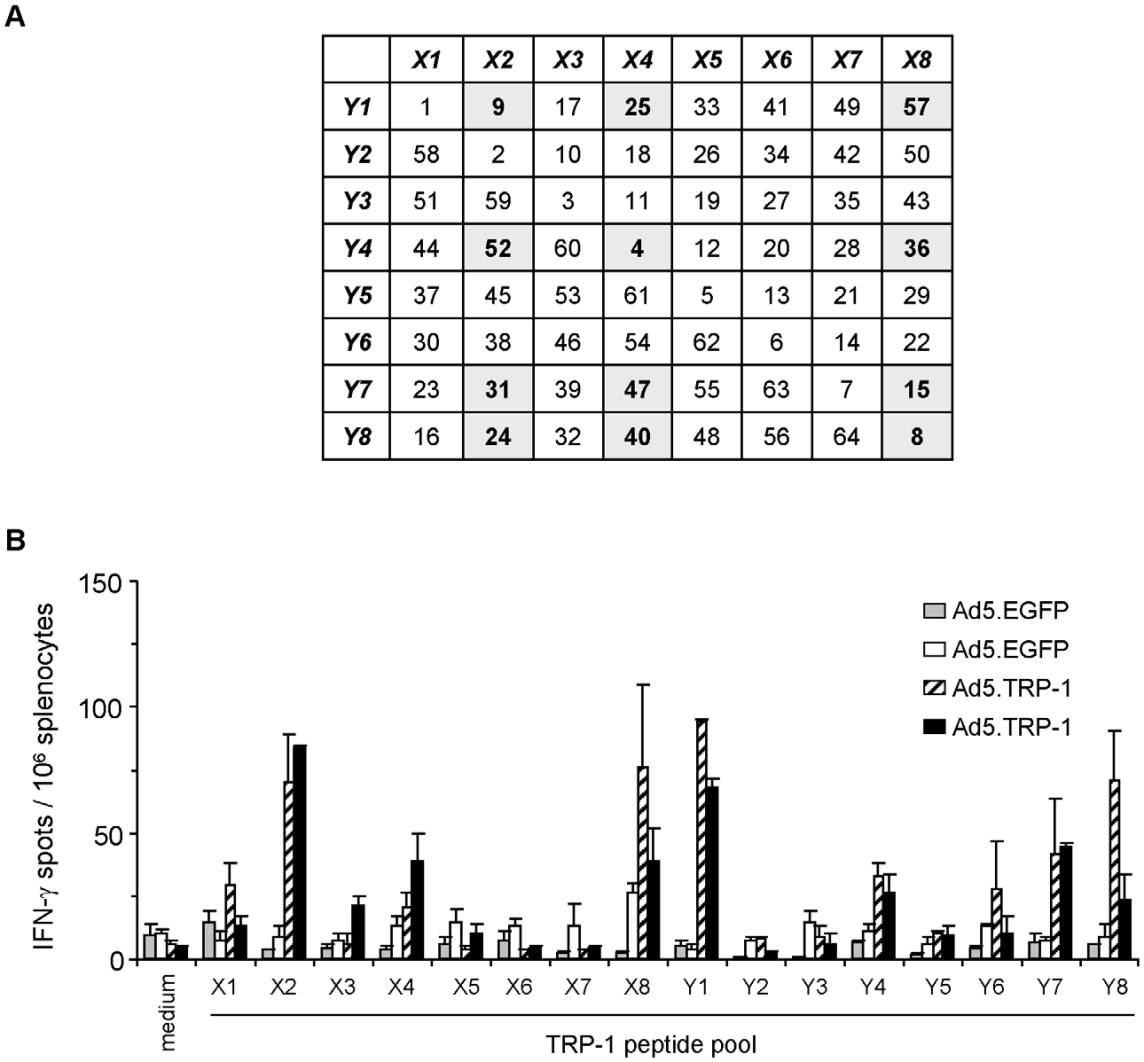
Combinatorial peptide library screening allows detection of individual library peptides containing TRP-1-specific T cell epitopes. *A*, Composition of the TRP-1-specific library peptide pools X1 to X8 and Y1 to Y8 used for combinatorial screening of specific T cell responses *ex vivo*. Individual peptides determined by combinatorial screening are highlighted. *B*, Spleen cells from HLA-DR3tg mice injected i.p. with 5×10^8^ pfu Ad5.TRP-1 or Ad5.EGFP (2 mice per group) were screened *ex vivo* by IFN-γ ELISpot assay for recognition of TRP-1-specific library peptide pools. T cell responses of two control mice (Ad5.EGFP) and two Ad5.TRP-1-immunized mice are represented as individual columns in the diagram. Error bars show standard error of the mean of duplicates. Experiments were performed four times, yielding similar results.

In addition to TRP-2 we applied the above described screening strategy also to the identification of CD4^+^ T cell epitopes from the differentiation antigen TRP-1. The new recombinant Ad5.TRP-1 mediated strong TRP-1 expression in infected HEK293T cells as confirmed by immunofluorescence microscopy using the TRP-1-specific monoclonal antibody TA99 (data not shown). This virus was then used for immunization of HLA-DR3tg mice whose splenocytes were subsequently tested for their reactivity against a TRP-1-specific peptide library consisting of 66 overlapping 20mers. Library peptides #1–#64 were combined in 16 pools (X1–X8 and Y1–Y8) as described for TRP-2 (Fig. 4A). The two additional peptides #65 and #66 were tested separately in initial screening experiments. However, since none of these two peptides elicited specific IFN-γ secretion among spleen cells (data not shown) they were excluded from further assays. Screening of the peptide library revealed that peptide pools X2, X4, X8 and Y1, Y4, Y7, Y8 specifically stimulated spleen cells of both Ad5.TRP-1-immunized mice but not of Ad5.EGFP-treated animals (Fig. 4B), leading to the selection of 12 single candidate peptides highlighted in Fig. 4A.

### Identification of TRP-1-derived CD4^+^ T cell epitopes in HLA-DR3tg mice

**Figure 5:**
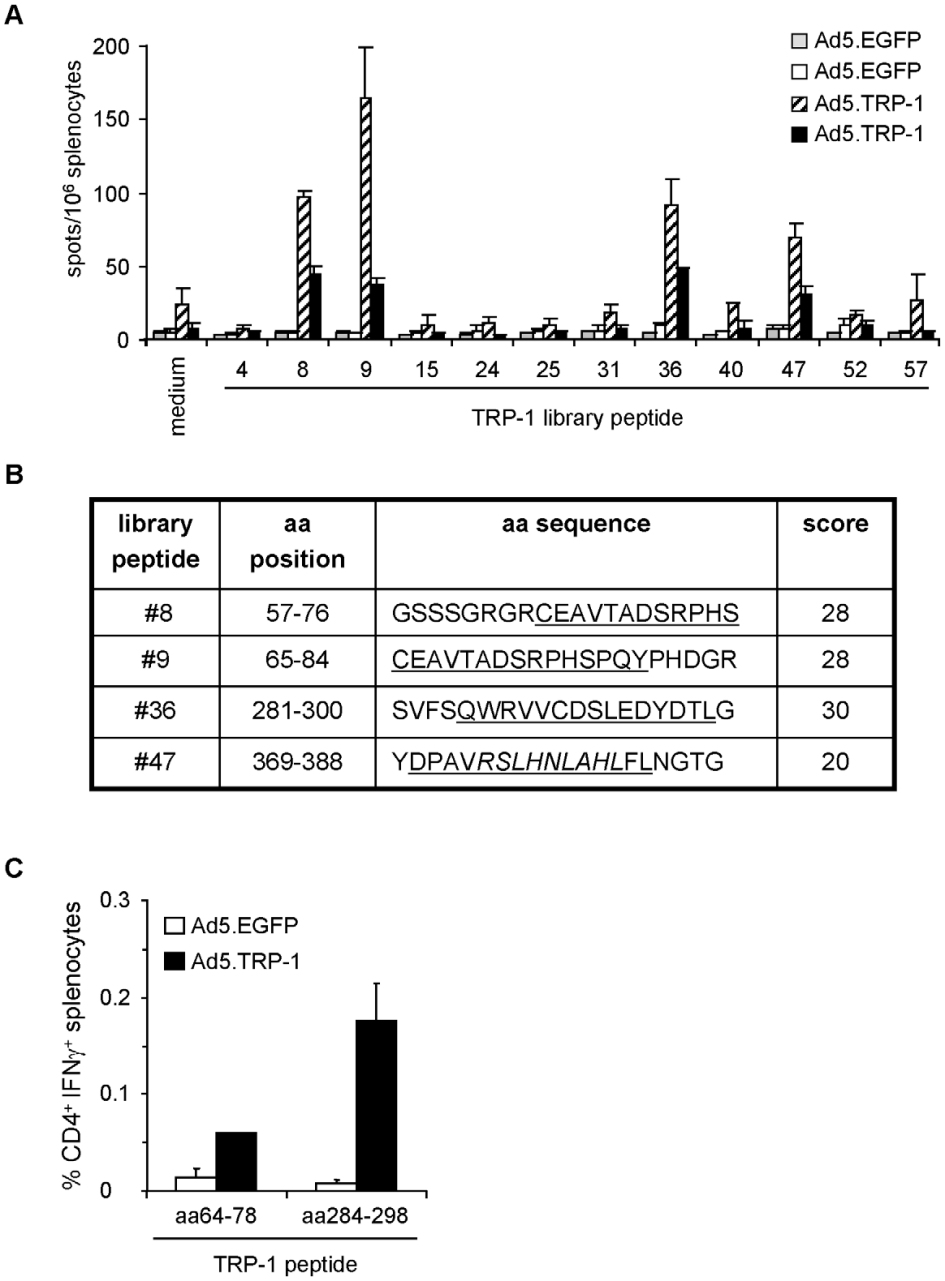
Phenotypic analysis of TRP-1-specific T cell responses induced in HLA-DR3tg mice upon immunization with Ad5.TRP-1. *A*, Spleen cells from HLA-DR3tg mice injected i.p. with 5×10^8^ pfu Ad5.TRP-1 or Ad5.EGFP (2 mice per group) were screened *ex vivo* by IFN-γ ELISpot assay for reactivity against selected TRP-1-derived library peptides. T cell responses of two control mice (Ad5.EGFP) and two Ad5.TRP-1-immunized mice are represented as individual columns in the diagram. Error bars show standard error of the mean of duplicates. Experiments were performed three times, yielding similar results. *B*, Selected TRP-1-derived library peptides #8, #9, #36 and #47 are indicated by amino acid (aa) positions and aa sequence. Peptides were analyzed *in silico* by the SYFPEITHI algorithm for the presence of potential HLA-DRB*0301 binding sequences (underlined) [33]. Prediction scores for HLA-DRB1*0301-restricted epitopes are listed on the right. The potential H2-restricted CTL epitope TRP-1_374–382_ is given in italics. *C*, Spleen cells of HLA-DR3tg mice injected i.p. with 5×10^8^ pfu Ad5.TRP-1 or Ad5.EGFP (3 mice per group) were analyzed for the presence of peptide-reactive T cells by intracellular IFN-γ staining after one round of *in vitro* re-stimulation with the indicated peptides. Note: The amino acid sequence of these peptides was shifted towards the N-terminus by one residue relative to the predicted epitope sequence. Stained cells were analyzed by flow cytometry for the percentage of IFN-γ^+^ CD4^+^ T cells. Error bars show standard error of the mean of three immunized mice. Experiments were performed twice yielding similar results.

Analysis of the individual candidates from the TRP-1 peptide library demonstrated that peptides #8, #9, #36 and #47 specifically induced cytokine release by splenocytes of Ad5.TRP-1-immunized HLA-DR3tg mice (Fig. 5A). Subsequent *in silico* analysis of these peptides predicted library peptide #9 to contain a HLA-DRB1*0301-restricted CD4^+^ T cell epitope encompassing TRP-1_65–79_ (Fig. 5B), partly present also within the sequence of library peptide #8. Within library peptide #36 the sequence TRP-1_285–299_ was predicted to represent a HLA-DRB1*0301-restricted CD4^+^ T cell epitope. Library peptide #47 was again an exceptional candidate predicted to contain a CD4^+^ T cell epitope (score 20) as well as a nonamer (TRP-1_374–382_) perfectly fitting the H2-D^b^ binding motif [35] with a score of 26 [33].

In order to define the T cell subset responding to the candidate peptides we performed intracellular IFN-γ stainings on spleen cells from HLA-DR3tg mice immunized with Ad5.TRP-1 or Ad5.EGFP. Stainings were performed after one round of *in vitro* restimulation with candidate peptide epitopes. In these assays the peptides TRP-1_64–78_ and TRP-1_284–298_ were used instead of TRP-1_65–78_ and TRP-1_285–298_. The shift in the amino acid sequences towards the N-terminus by one residue relative to the sequence predicted by SYFPEITHI, was performed in order to improve the peptide solubility without changing the natural sequence of the protein.

Using this strategy, we observed that splenocyte cultures from Ad5.TRP-1-immunized mice contained an average of 0.06% CD4^+^ IFN-γ^+^ cells responding to TRP-1_64–78_ versus 0.01% in case of Ad5.EGFP-treated mice (Fig. 5C). The responses against peptide TRP-1_284–298_ were much more pronounced. Cultures derived from Ad5.TRP-1-immunized mice harboured around 0.18% CD4^+^ IFN-γ^+^ splenocytes whereas less than 0.01% double-positive cells were detected in controls. No CD8^+^ T cell responses were observed against the two peptides. In contrast, CD8^+^ T cell reactivity was detectable with the candidate CTL-epitope TRP-1_374–382_ derived from library peptide #47 among spleen cells of Ad5.TRP-1-immunized mice (data not shown). Thus, the combinatorial screening approach could also be successfully applied for the identification of HLA-DRB1*0301-restricted CD4^+^ T cell epitopes derived from TRP-1.

### CD4^+^ T cells specific for TRP-1_284–298_ are present in PBMC from melanoma patients

**Figure 6:**
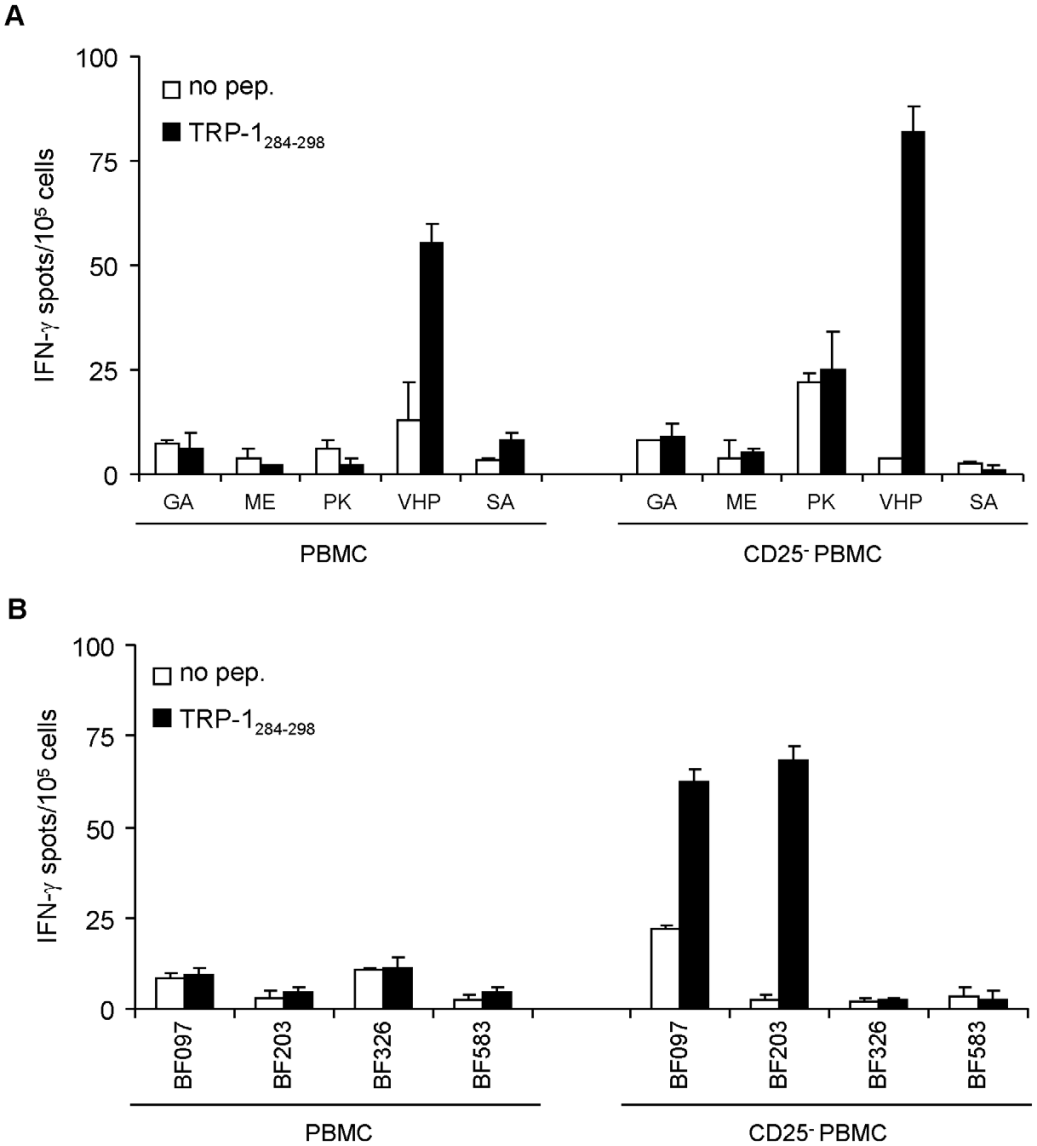
Detection of TRP-1_284–298_ reactive CD4^+^ T cells in melanoma patients. Total PBMC (left panel) or CD25^+^-depleted PBMC (right panel) from five HLA-DRB1*03^+^ melanoma patients (*A*) and four HLA-DRB1*0301^+^ healthy donors (*B*) were stimulated *in vitro* with peptide TRP-1_284–298_. After 25 days, PBMC were tested for their peptide reactivity by IFN-γ ELISpot assay. All determinations were performed at least in duplicates. The data are presented as mean numbers of IFN-γ spots per 10^5^ cells. Error bars show standard error of the mean.

In the next step we analyzed peripheral blood cells from HLA-DRB1*03^+^ melanoma patients and HLA-DRB1*0301^+^ normal donors for the presence of TRP-1_64–78_- and TRP-1_284–298_-specific CD4^+^ T cells. Therefore, PBMC either left untreated or depleted of CD25^+^ cells were stimulated once with the each peptide individually. After a 25 day short term culture, cells were harvested and analyzed for their peptide reactivity by IFN-γ ELISpot assay (Fig. 6). Under both conditions melanoma patient VHP showed TRP-1_284–298_-specific T cell reactivity (Fig. 6A). However, TRP-1_284–298_-specific T cell responses became detectable in healthy donors only among CD25-depleted PBMC (Fig. 6B). In contrast to TRP-1_284–298_, none of the cultures showed reactivity against peptide TRP-1_64–78_ (data not shown).

**Figure 7:**
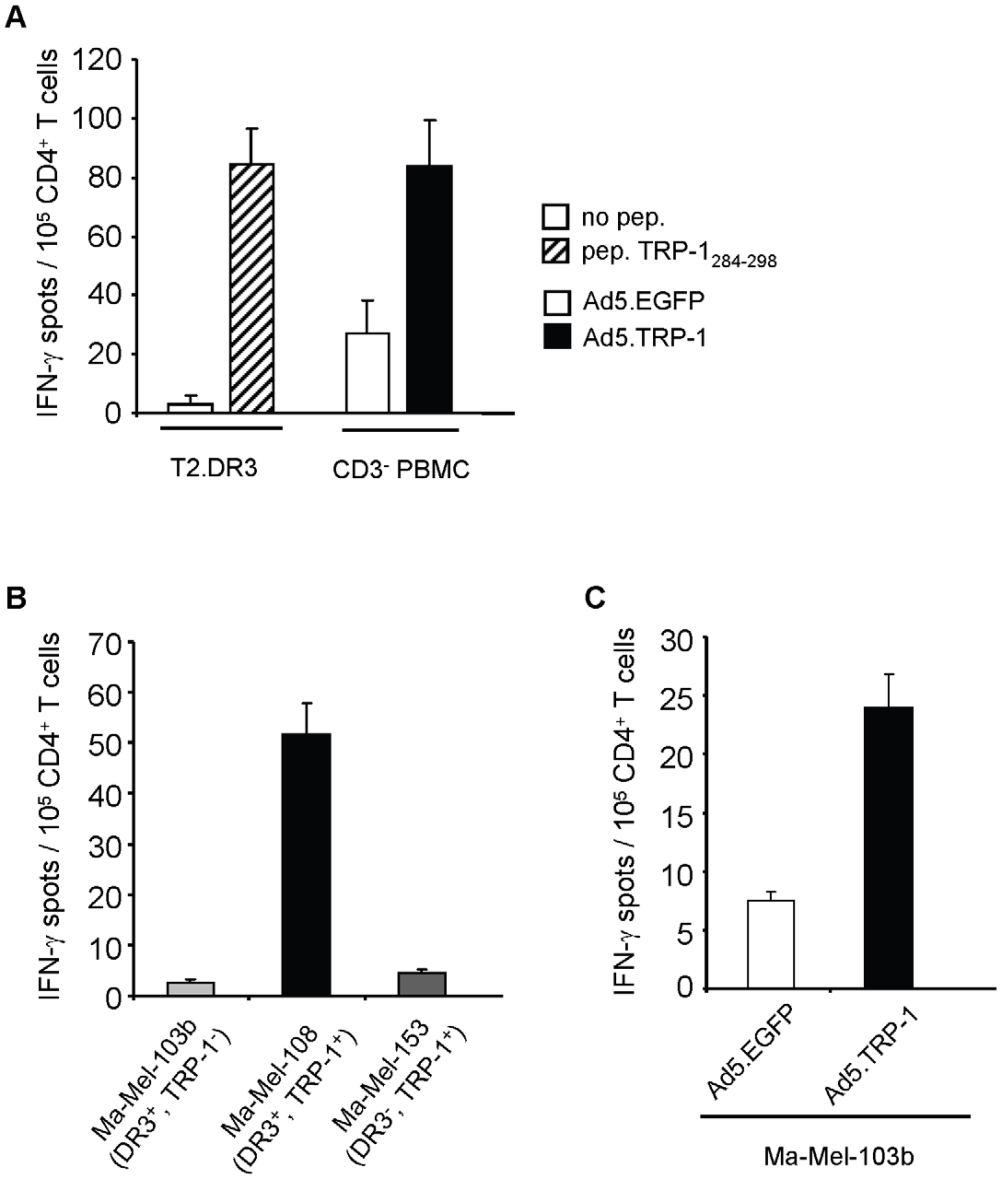
TRP-1_284–298_ represents a HLA-DRB1*0301-restricted CD4^+^ T cell epitope processed by human target cells. PBMC from melanoma patient VHP were stimulated once with peptide TRP-1_284–298_ (10 µg/ml). After 25 days, selected CD4^+^ T cells were tested for their reactivity against different target cells by IFN-γ ELISpot assay. *A*, Recognition of T2.DR3 target cells pulsed with peptide TRP-1_284–298_ and autologous CD3^+^-depleted PBMC (CD3^−^ PBMC) infected with Ad5.TRP-1 or with control virus is depicted. *B*, Reactivity of T cells against different melanoma cell lines Ma-Mel-103b (HLA-DRB1*0301^+^, TRP-1^−^), Ma-Mel-108 (HLA-DRB1*0301^+^, TRP-1^+^), Ma-Mel-153 (HLA-DRB1*0301^−^, TRP-1^+^) and *C*, against Ma-Mel-103b cells infected with Ad5.TRP-1 or control virus is presented. *A–C*, One representative out of two to three independent experiments is given. Error bars show standard error of the mean.

Finally, we set out to demonstrate the processing and presentation of TRP-1_284–298_ by human target cells. We obtained TRP-1_284–298_-specific CD4^+^ T cells after short term peptide stimulation of PBMC from patient VHP. Peptide-specificity and HLA-DRB1*0301-restricted activity of the CD4^+^ T cell line was demonstrated by the recognition of T2.DR3 target cells pulsed with antigenic peptide (Fig. 7A). By coincubation of TRP-1_284–298_-specific CD4^+^ T cells with infected autologous target cells, it turned out that Ad5.TRP-1-infected target cells, in contrast to Ad5.EGFP-infected control cells, strongly induced IFN-γ secretion among TRP-1_284–298_-specific CD4^+^ T cells, demonstrating processing and presentation of the respective epitope by human targets (Fig. 7A). In addition, the TRP-1_284–298_-specific CD4^+^ T cell line was used to investigate if human melanoma cell lines could serve as stimulatory targets. Among the melanoma cell lines tested we found that Ma-Mel-108 cells expressing both, HLA-DRB1*0301 and TRP-1, were recognized. In contrast, melanoma cells lines expressing HLA-DRB1*0301 while lacking TRP-1, like Ma-Mel-103b as well as HLA-DRB1*0301^−^ melanoma cell lines expressing TRP-1, such as Ma-Mel-153, were ignored (Fig. 7B). Notably, infection of melanoma cell line Ma-Mel-103b with Ad5.TRP-1 turned this cell line into a susceptible target for TRP-1_284–298_-specific CD4^+^ T cells (Fig. 7C). Taken together, these results demonstrate, that TRP-1_284–298_ can be presented by different HLA-DRB1*0301^+^, TRP1^+^ target cells, thus representing a new CD4^+^ T cell epitope.

## Discussion

The role of CD4^+^ T cells in anti-tumor immunity has long been restricted to their helper function in primary activation and maintenance of antigen-specific cytotoxic CD8^+^ T cell responses [17]–[19]. However, several mouse studies point to additional roles of CD4^+^ T cells in tumor control. Adoptively transferred tumor antigen-specific CD4^+^ T cells have been demonstrated to mediate dormancy or regression of tumors [12], [16], [21], even eradication of large established tumor masses was observed [11], [13], [14]. In several cases this was shown to be an indirect effect mediated by cytokines released from CD4^+^ T cells [12], [16] and by their interaction with other immune cells like macrophages and NK cells [12], [21]. However, studies by two independent groups demonstrated that murine CD4^+^ T cells specific for TRP-1, upon adoptive transfer into a lymphopenic host, differentiated into cytolytic, IFN-γ secreting effectors [11], [14] that directly killed MHC class II-positive B16 melanoma cells [11]. Interestingly, MHC class II surface expression on human melanoma cells has been described to be associated with good prognosis [36], suggesting that endogenous tumor antigen-specific CD4^+^ T cells might become activated by tumor cells and exert their anti-tumor effects directly or indirectly. Recently, the clinical relevance of adoptively transferred tumor antigen-specific CD4^+^ T cells was impressively documented in a report describing the complete remission of stage IV metastatic melanoma [15].

This obvious importance of CD4^+^ T cells in tumor control has strongly stimulated the interest in epitopes recognized by CD4^+^ T cells. We applied a comprehensive new methodology for epitope identification based on the immunization of HLA-DR3tg mice with recombinant adenovirus encoding human tumor antigens and subsequent screening of the T cell responses *in vitro* with the help of combinatorial antigen-specific peptide libraries. This approach allowed us to directly focus on epitope containing peptides within the peptide library, thereby avoiding unnecessary consideration of *in silico* predicted, but finally irrelevant candidates. Following this strategy we observed that Ad5.TRP-2 and Ad5.TRP-1 readily induced antigen-specific CD4^+^ T cell as well as CD8^+^ T cell responses, demonstrating that in principle our approach is suitable for the identification of MHC class I- and MHC class II-presented epitopes. Focusing on MHC class II, we identified TRP-2_60–74_ and TRP-2_149–163_ as target epitopes of CD4^+^ T cells in Ad5.TRP-2 immunized mice. We previously found TRP-2_60–74_ to be recognized also by CD4^+^ T cells from HLA-DR3tg mice injected with recombinant TRP-2 protein, indicating that T cell responses against this epitope can be elicited by different antigen formats [24]. Importantly, we confirmed TRP-2_149–163_ to be naturally processed and presented also in the human system, as already shown for TRP-2_60–74_ [24], and we could detect epitope-specific CD4^+^ T cells among PBMC of both, melanoma patients and healthy individuals. In case of TRP-1 we identified TRP-1_64–78_ and TRP-1_284–298_ as epitopes in HLA-DR3tg mice. We could detect TRP-1_284–298_–specific CD4^+^ T cell responses also in the human system, both among healthy donors and melanoma patients. However the frequency of responding patients was low (20%, Fig. 6A) and healthy donors showed reactivity against this epitope only upon depletion of CD25^+^ T cells, albeit only in two out of four individuals. We have not investigated the reasons why only a minor fraction of individuals responded to this epitope, but it appears conceivable that TRP-1_284–298_–specific CD4^+^ T cell responses being potentially self-reactive might be kept in check by peripheral tolerance mechanisms, such as Tregs, as described below. In the case of the epitope TRP-1_64–78_ we failed to detect specific CD4^+^ T cell reactivity, both in patients and in healthy donors. One possibility for this observation might be deletion of TRP-1_64–78_-specific T cells by central or peripheral tolerance. Alternatively, the frequency of TRP-1_64–78_-specific T cells might have been too low for detection by the applied techniques. Moreover, it cannot be excluded that TRP-1_64–78_-specific T cells might actually be present which, however, secrete cytokines other than IFN-γ, like immunosuppressive IL-10 or TGF-β.

Interestingly, while T cell responses specific for TRP-1_284–298_ and TRP-2_149–163_ could be detected after short term peptide stimulation in whole PBMC populations from patients, healthy donors showed specific T cell reactivity only after depletion of CD25^+^ T cells. This is in accordance with previous studies demonstrating that naïve antigen-specific CD4^+^ T cells from healthy donors are silenced by CD25^+^ Treg cells while antigen-experienced CD4^+^ T cells of the memory phenotype, as they are likely to be present also within whole PBMC from our patient group, are less sensitive to suppression [37], [38]. However, even after CD25^+^ Treg depletion, T cells specific for TRP-1_64–78_ were neither detected in PBMC from healthy individuals nor from patients, which might be due to the above indicated reasons. On the other hand, HLA-DR3tg mice, after immunization with Ad5.TRP-1, as performed in our study, might be a source of TRP-1_64–78_-specific T cells, whose T cell receptors (TCR) could be cloned to generate TCR-recombinant tumor-reactive CD4^+^ T cells for adoptive therapy. In case of CD8^+^ T cells, this approach has already been followed by Johnson et al., who cloned from CD8^+^ T cells of HLA-A*02-transgenic mice highly avid TCR recognizing the human melanoma differentiation antigen gp100, in order to generate recombinant human PBL for adoptive cellular therapy of melanoma patients [39].

Collectively, we demonstrate for the first time that by strategic combination of HLA class II-transgenic mice, immunization with recombinant Ad5 and combinatorial antigen-specific peptide library screening, CD4^+^ T cell epitopes from melanoma differentiation antigens can readily be defined, pointing to a broad applicability of this approach to target antigens of other tumor entities and to different HLA class II molecules even without prior characterization of their peptide binding motives.
